# Predictors of time to recovery of preterm neonates with respiratory distress syndrome admitted in University of Gondar comprehensive specialized hospital neonatal intensive care unit North West Ethiopia

**DOI:** 10.1371/journal.pone.0275366

**Published:** 2022-10-14

**Authors:** Ayenew Engida Yismaw, Abebaw Addis Gelagay, Malede Mequanent Sisay, Yazacchew Engida Yismaw

**Affiliations:** 1 Department of Clinical Midwifery School of Midwifery, College of Medicine and Health Science, University of Gondar, Gondar, Ethiopia; 2 Department of Reproductive Health, Institute of Public Health, College of Medicine and Health Science, University of Gondar, Gondar, Ethiopia; 3 Department of Epidemiology and Biostatistics, Institute of Public Health, College of Medicine and Health Science, University of Gondar, Gondar, Ethiopia; 4 Department of Pharmacy, School of Health Science, College of Medicine and Health Science, Bahir Dar University, Bahir Dar, Ethiopia; University of the Witwatersrand Faculty of Health Sciences / Pan African University, SOUTH AFRICA

## Abstract

**Background:**

Respiratory distress syndrome (RDS) is caused by a deficiency of a molecule called surfactant. It occurs in newborns born before 37 weeks of gestation. It is a main cause of morbidity and mortality in the early neonatal period.

Therefore, this study aims to assess median time to recovery and predictors of preterm neonates with respiratory distress syndrome admitted in University of Gondar comprehensive specialized hospital Northwest Ethiopia 2020.

**Methods:**

Institution based retrospective follow up study was conducted on 386 preterm neonates with hyaline membrane disease who were admitted in the neonatal intensive care unit from January, 2016 to December 2018. The data were entered in to EPI info version 7.0 and transferred to Stata version 14.0 for analysis. Both bi-variable and multi variable Weibull parametric model were fitted to identify predictors with 95% confidence interval of hazard ratio (HR) and p-value. P-value less than 0.05 in the multivariable model showed the presence of significant association between covariates and the dependent variable.

**Results:**

The overall median length of recovery were 11 day with an interquartile range of (7, 16) neonate-days. Being a product of multiple pregnancy (AHR 1.67; 95%CI (1.25, 2.23)), vaginal mode of delivery (AHR 1.6; 95%CI (1.13, 2.26)), and neonatal hypothermia at admission (AHR 1.6; 95%CI (1.13, 2.26)) were found to be significant predictors of time to recovery.

**Conclusion:**

In this study the median time to recovery of preterm neonates with respiratory distress syndrome was slower than the clinical recommendations. Receiving bag and mask ventilation and hypothermia decreased the recovery whereas, vaginal delivery gestational age at birth, being multiple pregnancy, birth weight ≥2000grams were enhance the recovery of preterm neonates with RDS.

## Introduction

World health organization defined preterm birth as birth after 28 weeks and before 37 completed weeks of gestation since last menstrual period of a woman [1]. About 10% of births, are preterm each year in the world. It is the most common cause of newborn deaths accounting for 35% [2]. About 28% of neonatal death within seventh day of life are due to preterm birth [3].

Globally the leading cause for neonatal intensive care (NICU) admission is preterm and more than 60% of prematurity occurs in low- and middle-income countries (LMICs) [4, 5]. Studies conducted on preterm birth recommends that focused and continuous studies should be conducted to fill the information gap in many developing countries [6].

The United Nations of children’s fund reported that, in Ethiopia preterm is one of the main causes of neonatal hospital admission and death accounting for 23% of all other causes of neonatal death [7]. Causal factors to preterm birth are multifactorial involving maternal, fetal, genetically, environmental exposure, behavioral and socio-economic factors as well as iatrogenic prematurity [8, 9].

Even if preterm birth is not an acute disease, it is the major causes of infants’ death and continues to be significant public health problem by increasing the economic expenditure to health system of a developing nation [10].

Among the complications of preterm neonates, respiratory distress syndrome (RDS) is the major one which is induced by the shortage or deficiency of surfactant in the lungs with the clinical manifestations of grunting, tachypnea, retractions, nasal flaring and increased the effort of inhalation at birth, or in a while subsequently and a necessity for complementary oxygen support [11]. It is the main cause of morbidity and mortality that accounts 7%–50% of preterm newborns [12].

The overall incidence of respiratory distress syndrome is 10–15%, but the prevalence decreases with increasing maturity. It may occur among 60% of babies with 28–32 weeks of gestation, 15–30% of those between 32-36weeks, and in about 5% beyond 37 weeks of gestation [13, 14].

A hospital-based studies conducted in India among neonates admitted to NICU due to respiratory distress syndrome reported that the recovery rate was account about 64.5 percent [15, 16]. A cross-sectional study conducted in Assiut University Children Hospital, Egypt on respiratory distress and its outcome among neonates admitted in NICU reported that 30.5% [17], in (NICU) of Fauji Foundation hospital Rawalpindi 62.86% [18] and Lautech Teaching Hospital, Osogbo, Nigeria reported that 59.79% were recovered and discharged to home [19].

A retrospective cohort study conducted in NICU of a University Teaching and Referral Hospital in Southern Ethiopia reported that 34.04% [20] and in Jimma University Specialized Hospital NICU reported that 42.78% of neonates with RDS recovered [21].

Studies done in Johannesburg, South Africa [22], and Jimma, Ethiopia [20] reported that place of birth were associated with preterm neonatal recovery. Studies showed that multiple pregnancies were a significant factor for the recovery of preterm neonates with RDS [20, 23]. Studies conducted in different settings reported that neonatal sex, weight at specific gestational age and birth weight were the main significant predictor for recovery of preterm neonates with RDS [20–22]. In other studies gestational age (GA) at birth, mode of delivery and Apgar score at first and fifth minutes were significant factors for recovery of preterm neonates with RDS [21, 24–26].

Studies in different settings reported that the presence of neonatal clinical problems such as perinatal asphyxia (PNA), jaundice, hypothermia, hypoglycemia and neonatal sepsis were strong predictors for recovery of preterm neonates with RDS [21, 24, 26].

Studies reported that Nasal continuous positive air pressure (NCPAP) and kangaroo mother care (KMC) were reported as determinant factors for recovery of preterm neonates with RDS [21, 22, 27].

Findings addressing multiple risk factors aimed at reducing the risk of prematurity and address the survival gap of premature babies to achieve sustainable development Goal 3.2 entitled end preventable deaths of newborns and under-five children by 2030 [15].

Though studies have been conducted on neonatal mortality in general, however no study has been done regarding preterm neonates with RDS and the complications following it in Ethiopia. Therefore, this study aimed to determine the time to recovery and predictors of preterm neonates with RDS and will serve as baseline for further research. This research will also help to evaluate the clinical service and be used to inform respective clinicians and stakeholders about the burden of prematurity, and its complications to assist in planning for the possible need of action.

## Methods

### Study design and area

Facility-based retrospective follow up study was conducted among preterm neonates admitted with a diagnosis of RDS in NICU of the University of Gondar comprehensive specialized hospital Northwest Ethiopia from January 2017 to December, 2019. The hospital is one of the largest teaching hospitals in the Amhara region providing tertiary level care for more than seven million people in the northwest part of the country. It is located at 727 km and 180 km far from Addis Ababa and Bahir Dar, the capital city of Ethiopia and the Amhara region to Northwest respectively. Neonatology is a unit under the pediatrics and child health department. It provides an outpatient and inpatient medical service for neonates. Neonatal intensive care unit particularly offers intensive care for neonates on an inpatient basis.

### Source and study populations

The source population were all preterm neonates who were admitted to the Neonatal Intensive Care Unit with a diagnosis of RDS at University of Gondar Comprehensive Specialized Hospital.

The study population included all preterm neonates who were admitted to (NICU) with a diagnosis of RDS at University of Gondar comprehensive specialized Hospital from January 2017 to December 2019.

### Sample size determination and sampling procedures

The sample size was determined by using survival analysis formula based on the following assumptions; number of event (E), probability of the event (d) and values of Z_α/2_ and Z*β* as 1.96& 0.842 at 95% confidence level respectively. Where q_1_ was probability of event in previous studies and q_0_ = 1- q_1._

E=Zα/2+Zβ^2logHR2q1q0

[28]

Then the sample size

$$N=\frac{E}{d}$$

According to a retrospective follow up study to model survival probability of premature infants who were under follow-up and identify significant risk factors revealed that recovery of preterm neonates with RDS was 42.78% [21] which gave the final total sample size of 398. However, we utilized the available records of all preterm neonates with RDS during the study period which were 386.

### Data collection procedures and quality control

Before data collection, the records were reviewed and preterm neonatal cards were identified by their medical registration/card number. Then data collectors reviewed and extracted data from patients’ charts and registries using a structured check list. Data extraction check-lists was prepared in English from Health and Medical Information System (HMIS) registration format and patient’s card. Three BSc midwife professionals collected the data with the supervision of the principal investigator. The patients’ card clerks also supported them by identifying the cards of neonates. Training of the objective of the study and how to review the documents as per the data extraction format was conducted. The PI was supervise the overall process. The filled formats were checked for completeness by the PI supervisors.

### Data processing and analysis

The data were entered in to EPI info version 7 and exported to Stata 14 statistical software for further analysis. Descriptive summary statistics like median survival time, Kaplan Meier survival estimation curve and Log rank test were computed. Proportional hazard assumption (PHA) was checked both graphically and hypothesis test called Schoenfeld residual test. At the same time Schoenfeld’s residual test was done for almost all variables and met proportional hazard assumption. Schoenfeld residuals test (global test) showed that proportional hazard assumption PHA was satisfied.

Then, parametric models were done for time to recovery of preterm neonates with respiratory distress syndrome to identify the potential determinants.

Hazard ratio is used as a measure of probability of recovery, assuming that the Survival model is usually expressed in terms of hazard function.

Once Cox regression model fitted, parametric survival analysis models were fitted by considering the baseline hazard with different distribution assumption. Under the parametric approach, the baseline hazard is defined as a parametric function and the vector of its parameters are estimated together with the regression coefficients.

A more parsimonious hazard model was chosen by means of the log likelihood ratio (LR) test and Akaike Information Criterion (AIC). The best fitted model was chosen using AIC and those having the smallest AIC were considered as a best fitted model. Similarly, goodness of model fitness also checked using Cox Snell residual test.

## Ethical approval

Ethical approval was obtained from Institutional Review Board (IRB) of Institute of Public Health, CMHS, University of Gondar. The IRB of Institute of Public health, College of Medicine and health Sciences (CMHS) has waved for informed consent of the medical records of participants. Permission letter was obtained from the hospital administration and names of patients were not be included during data collection.

## Results

### Characteristics of the preterm neonates

Among 386 preterm neonates with RDS 229 (59.3%) were males, 239 (61.9%) were singleton pregnancy. About 109 (28.24%) of neonates have Apgar score <4 with in the first five minute and 176(45.6%) required a bag and mask ventilation immediately after delivery (Table 1).

**Table 1:** Characteristics of preterm neonates admitted in NICU at University of Gondar comprehensive specialized hospital with a diagnosis of RDS from January 2016 to December 2018 (n = 386).

Characteristics	Frequency	Percent
Place of birth		
Home	17	4.41
Health center	92	23.83
Hospital	277	71.76
Type of pregnancy		
Singleton	239	61.92
Multiple	147	38.08
Mode of delivery		
Spontaneous vaginal delivery	287	74.35
Caesarean section	90	23.32
Instrument assisted delivery	9	2.33
Sex of the neonate		
Male	229	59.33
Female	157	40.67
Gestational age		
< 32 weeks	98	25.39
32–35 weeks	211	54.66
35–37 weeks	77	19.95
Weight for gestational age at birth		
Small	82	21.24
Appropriate	304	78.76
Newborn cry immediately at birth		
Yes	277	71.76
No	109	28.24
Bag and mask resuscitation at birth		
Yes	176	45.60
No	210	54.40
Perinatal asphyxia diagnosed at birth		
Yes	119	30.83
No	267	69.17
Hypothermia diagnosed at admission		
Yes	322	83.42
No	64	16.58
Hypoglycemia diagnosed at admission		
Yes	82	21.24
No	304	78.76
Jaundice		
Yes	102	26.42
No	284	73.58
Newborn diagnosed with clinical sepsis		
Yes	299	77.46
No	87	22.54
Neonate received continuous positive airway pressure		
Yes	196	50.78
No	190	49.22
Newborn received kangaroo mother care		
Yes	45	11.66
No	341	88.34

### Cumulative incidence of recovery of preterm neonates with RDS

In this study the cumulative incidence of recovery of preterm neonates with RDS was 60.9 (95%CI; (55.9, 65.7)) percent.

### Time to recovery of preterm neonates with RDS admitted to NICU

The overall median length of hospital stay for preterm neonates under the study was 7 days, which gave a total of 3511 neonate-days observation and median length of recovery were 11 neonate-days with an interquartile range of (7, 16) neonate-days.

**Fig 1:**
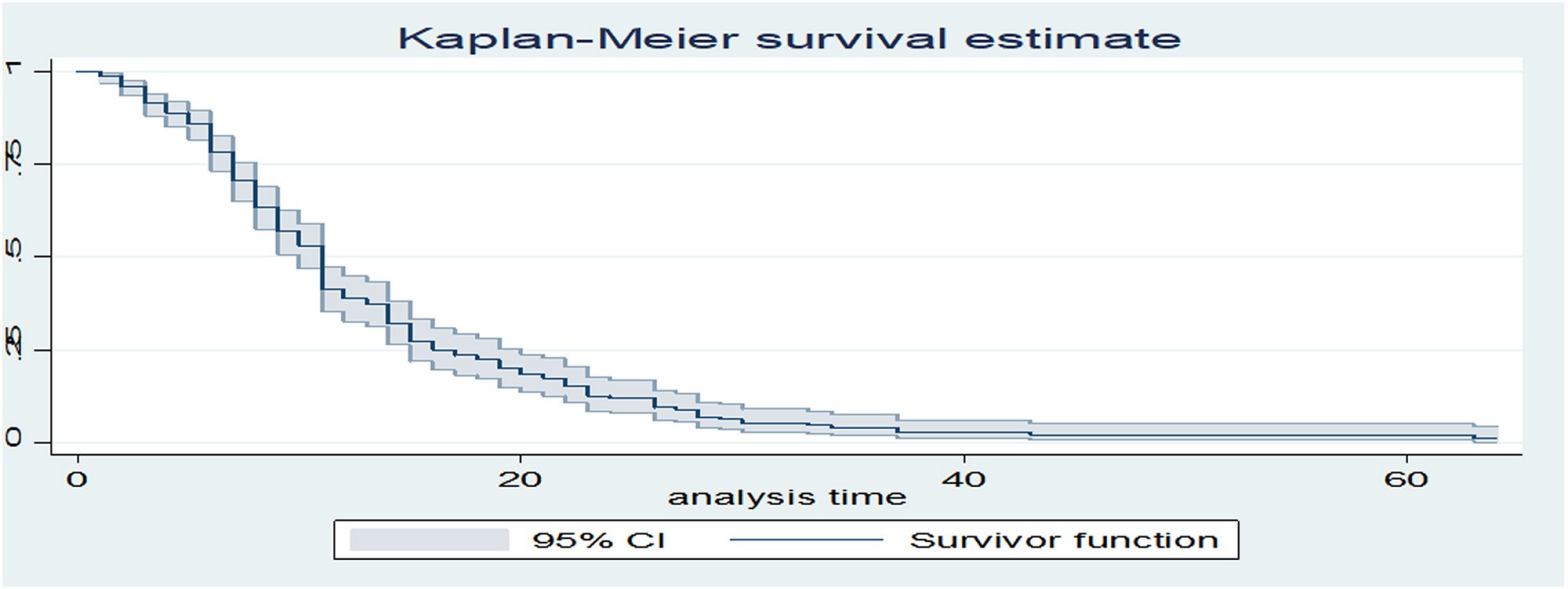
Kaplan-Meier curve of surviving preterm neonates with RDS admitted in NICU at University of Gondar comprehensive specialized hospital from January 2016 to December 2018 (n = 386).

The cumulative probability of recovery at the end of seventh days was 79.6%, at twenty-eight days were 9.2% (Table 2 and Fig 1).

**Table 2:** Recovery probability of preterm neonates with RDS admitted in NICU at University of Gondar comprehensive specialized hospital from January 2016 to December 2018 (n = 386).

Time interval in days	Total at beginning	Recovery	Survival probability %	95% CI
Within 24 hours	386	5	95.34	92.7, 97.04
(1–7)	363	61	79.57	74.73, 83.58
(7–14)	206	99	38.54	32.53, 44.51
(14–21)	79	39	18.89	13.98, 24.38
(21–28)	35	18	9.18	5.67, 13.70
28+	17	13	1.22	0.25, 3.95

### Model diagnosis and adequacy tests

Differences in all key variables between strata were determined using log rank (χ2) test and assessed the equality of hazard for the different explanatory variables.

**Fig 2:**
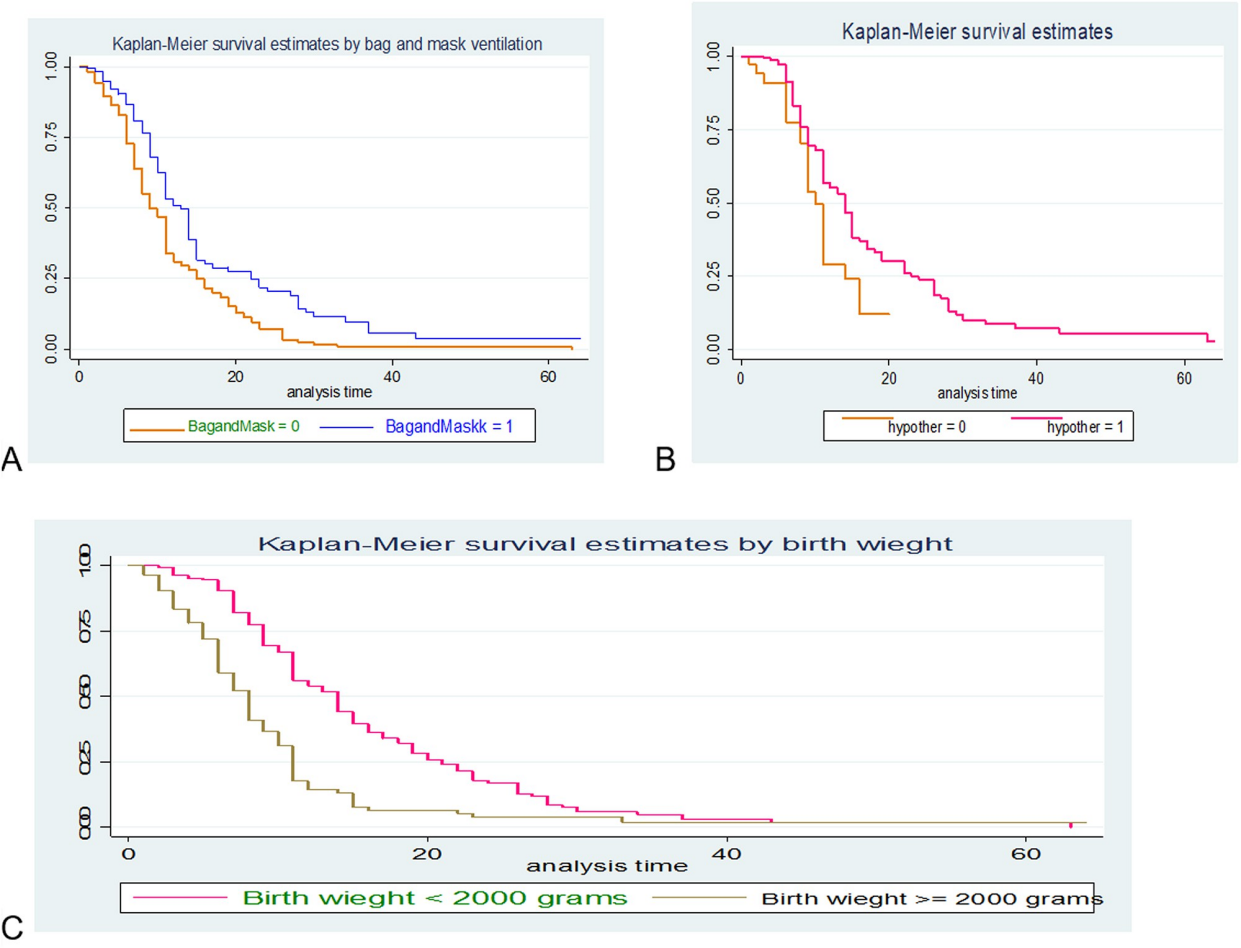
Kaplan-Meier curve of survival of preterm neonates with RDS admitted in NICU by bag & mask ventilation (A), hypothermia (B) and birth weight (C) at University of Gondar comprehensive specialized hospital from January 2016 to December 2018 (n = 386).

Similarly the plot Kaplan Meier survival by taking bag and mask ventilation (A), hypothermia (B) and birth weight (C) against survival time were done and the study indicated roughly parallel graphs for duration of follow up, thus proportional hazard assumption was met (Fig 2).

### Goodness of fit test

**Fig 3:**
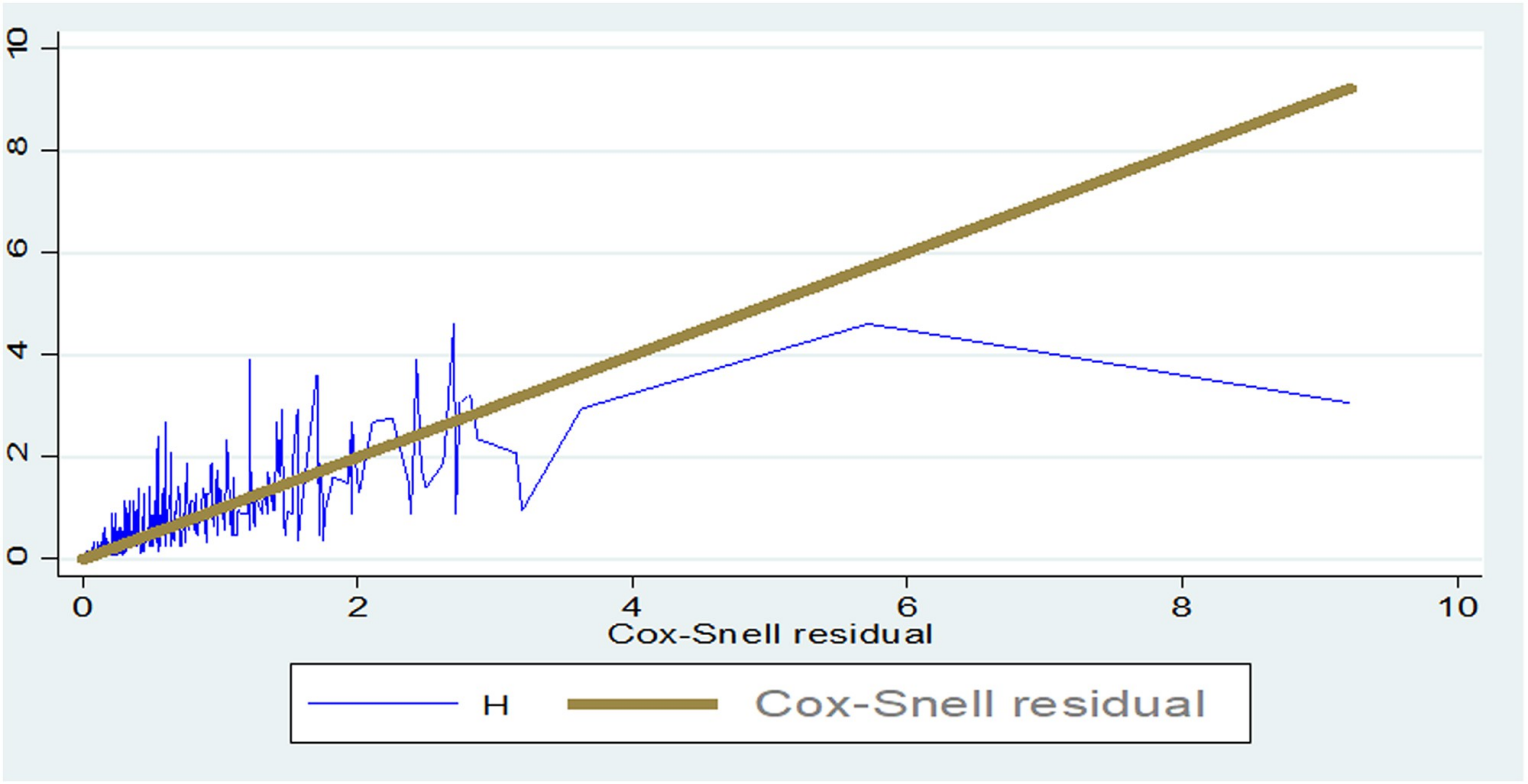
Goodness of fit test for univariate distribution of Weibull hazard model.

The goodness of fit for the fitted model also performed using the Cox Snell residual test and the model was adequate (Fig 3).

### Factors associated with the recovery of preterm neonates with RDS

Univariable and multivariable Weibull parametric model was fitted to identify determinants of time to recovery for preterm neonates with RDS from admission to discharge in the neonatal intensive care unit.

Findings from the bivariable analysis showed that gestational age, birth weight, neonatal hypothermia at admission, and neonate taking bag &mask ventilation were significantly associated with time to recovery of preterm neonates with RDS.

However, in the multi-variable analysis type of pregnancy, mode of delivery, gestational age, birth weight, neonatal hypothermia at admission, and neonate taking bag &mask ventilation have remained statistically significant for time to recovery of preterm neonates with RDS (Table 3).

**Table 3:** Weibull hazard model for predictors of time to recovery among preterm neonates with RDS admitted in NICU at University of Gondar comprehensive specialized hospital from January 2016 to December 2018 (n = 386).

Predictor Variables	Event	Censored	CHR (95% CI)	AHR (95% CI)
**Place of delivery**				
Home	7	10	**1**	1
Health Facility	228	141	0.62(0.29, 1.32)	1.19(0.52, 2.74)
**Type of pregnancy**				
Singleton	**135**	**104**	**1**	**1**
Multiple	**100**	**47**	**1.1(0.85, 1.42)**	**1.67(1.25, 2.23)** **
**Mode of delivery**				
Vaginal delivery	**178**	**109**	**1.32(0.98, 1.78)**	**1.6(1.13, 2.26)** *
Cesarean delivery	**57**	**42**	**1**	**1**
**Gestational age**			**1.30(1.20,1.40)**	**1.26(1.15, 1.37)** **
**Birth weight**				
**<2000gram**	**136**	**110**	**1**	**1**
≥ **2000gram**	**99**	**41**	**2.22(1.71, 2.88)**	**2.12(1.55, 2.89)** **
**Sex of neonate**				
Male	140	89	1	1
Female	95	62	0.99(0.76, 1.28)	1.12(0.84, 1.48)
**Neonatal hypothermia at admission**				
No	**47**	**17**	**1**	**1**
Yes	**188**	**134**	**0.58(0.42, 0.80)**	**0.55 (0.39, 0.78)** **
**Neonate diagnosed with jaundice**				
No	180	104	**1**	1
Yes	55	47	0.96(0.70, 1.30)	1.02 (0.68, 1.54)
**Neonate received KMC**				
No	197	144	1	1
Yes	38	7	0.97(0.69, 1.37)	1.24 (0.86, 1.79)
**Neonate received bag &mask ventilation**				
No	**151**	**59**	**1**	**1**
Yes	**84**	**92**	**0.56(0.43, 0.73)**	**0.54(0.35, 0.82)** **
**Neonate taking NCPAP**				
No	51	100	1	**1**
Yes	145	90	0.95(0.73, 1.23)	0.96(0.73, 1.27)

NB:

* P-value < 0.05,

** P-value <0.005

## Discussion

In this study the median time and cumulative incidence of recovery of preterm neonates with RDS was 11 days and 60.9% respectively.

The median time to recovery of preterm neonates with RDS were 11 neonate-days with an interquartile range of (7, 16) neonate-days. This finding shows slow recovery as compared with different medical resources like up todate 20.3 and Medscape which said that with good medical treatment they should recover within 72 hours or 3 days. This might indicate that the treatment given in the hospital was not satisfactory.

The cumulative incidence of recovery of preterm neonates with RDS admitted in NICU of the University of Gondar comprehensive specialized hospital was 60.9% (95%CI;(55.90, 65.65)). This finding is in line with the findings reported by a study done in Lautech Teaching Hospital, Osogbo, Nigeria 59.79% [19], in Fauji Foundation hospital, Rawalpindi 62.86% [18] and in the NICU of MBGH RNT Medical College, India 64.5% [16].

Our study finding was higher than a study conducted in Jimma University Specialized Hospital, Jimma Ethiopia 42.78% [21], referral Hospital in Southern Ethiopia 34.04% [20] and Assiut University Children Hospital, Egypt 30.5% [17]. This might be due to the attention given by the government and none governmental organizations to improve health care services, equipped with skilled birth attendants, expansion of NICU in a well-organized manner and increased health seeking and utilization of the community to reduce neonatal morbidity and mortality, for the achievement of SDG.

In this study the recovery of preterm neonates with RDS who was delivered by vaginal delivery was 1.6 times more likely as compared with those delivered by cesarean delivery. This is clinically evidenced that, as the neonate is delivered through the vaginal birth canal, there is a pressure exerted on the chest to remove the fluid filling the neonatal lung leaving space for entry of air as well as better stimulate cardiorespiratory system.

Here in this study gestational age was a predictor variable for time to recovery of preterm neonate with RDS. A week increase in gestational age enhances the recovery of preterm neonates with RDS by 1.26 times. This finding was in line with a study conducted in Fauji Foundation hospital, Rawalpindi [18], Jimma University specialized hospital, Ethiopia [21] and prospective cohort study conducted in Addis Ababa St Paul’s Hospital Millennium Medical College [29].

This is because as the gestational age increase the neonate has better capability of producing surfactants for maximizing lung maturity and maintaining their cardiopulmonary system.

Preterm neonates with RDS who had comorbidity of hypothermia at admission had statistically significant effect on time to recovery and decreased by 45% as compared to those who had no hypothermia. The negative association between time to recovery of preterm neonates with RDS and hypothermia in our study is in line with studies conducted in Jimma University specialized hospital, Ethiopia [21] and Mt. Hope Women’s Hospital in Trinidad and Tobago [24]. This is because, hypothermia is clinically a known independent risk affecting the survival of preterm neonates’ and when appear as comorbidity with respiratory distress syndrome it affects the recovery more than expected.

A recovery of preterm neonate with RDS taking bag and mask ventilation has decreased by 46% as compared with those who hadn’t taking bag and mask ventilation. This might be due to the pressure exerted to the bag which may be beyond the capacity of the neonates’ lung and leading to alveolar damage causing life threatening complication.

In this study being multiple pregnancy has 1.67 times higher recovery among preterm neonates with RDS as compared to singleton pregnancy. This might be due to the reason that multiple pregnancies matured earlier than singleton pregnancies since the intrauterine live becomes worse and compromised starting 30 weeks of gestation onwards.

Birth weight greater or equal to 2000 grams among preterm neonates with RDS has 2.12 times higher recovery as compared with birth weight less than 2000grames. This is clinically evidenced that birth weight is another indicator for sign of maturity and copying life threatening complications.

## Conclusions

In this study the median time to recovery of preterm neonates with RDS was slow. In this study the cumulative incidence of recovery of preterm neonates with RDS was low as compared to clinical recommendations.

Receiving bag and mask ventilation and hypothermia as a comorbid condition at admission decreased the hazards of recovery of preterm neonates with RDS whereas, vaginal mode of delivery gestational age at birth, being multiple pregnancy, birth weight 2000grams and above were enhance the hazards of recovery of preterm neonates with (RDS). It is better to access NICU infrastructures and skilled manpower at primary health care institutions (primary hospitals, health centers) and give special attention for preterm neonates in the NICU to prevent comorbid complications like hypothermia.

## Limitation of the study

Since the data were secondary source it had missing information of some important variables which might have a significant association with outcome variable like maternal socio-demographic variables, obstetric conditions, immediate newborn care. Due to being secondary data it is unable to assess the quality of care provided which might have significant effect.

## Supporting information

S1 Data (DTA)

## Abbreviations

AHR: Adjusted Hazard Ratio
ANC: Antenatal Care
APGAR: Appearance Pulse Grimace Activity Respiration
APH: Antepartum Hemorrhage
BSc: Bachelors of Science
CI: Confidence Interval
EDHS: Ethiopian Demographic and Health Survey
GA: Gestational Age
HIV: Human Immune-Deficiency Virus
HR: Hazard Ratio
IRB: Institutional Review Board
KMC: Kangaroo Mother Care
NCPAP: Nasal Continuous Positive Air pressure
NICU: Neonatal Intensive Care Unit
NMR: Neonatal Mortality Rate
PHA: Proportional Hazard Assumption
PIH: Pregnancy Induced Hypertension
PNA: Perinatal Asphyxia
PROM: Prelabour Rupture of Membrane
PT: Preterm
RDS: Respiratory Distress Syndrome
UNICEF: United Nations International Emergency Children Fund
WHO: World Health Organization
